# Characterization of Dog Glutathione Transferase P1-1, an Enzyme Relevant to Veterinary Medicine

**DOI:** 10.3390/ijms22084079

**Published:** 2021-04-15

**Authors:** Aram Ismail, Elizabeth Lewis, Birgitta Sjödin, Bengt Mannervik

**Affiliations:** 1 Department of Biochemistry and Biophysics, Arrhenius Laboratories, Stockholm University, SE-10691 Stockholm, Sweden; aram.ismail@dbb.su.se (A.I.); birgitta.sjodin@dbb.su.se (B.S.); 2 College of Liberal Arts & Sciences, University of Illinois Urbana-Champaign, Champaign, IL 61801, USA; emlewis2@illinois.edu

**Keywords:** Telcyta, veterinary medicine, enzyme-activated chemotherapy, prodrugs, dog GST P1-1

## Abstract

Glutathione transferases (GSTs) form a family of detoxication enzymes instrumental in the inactivation and elimination of electrophilic mutagenic and carcinogenic compounds. The Pi class GST P1-1 is present in most tissues and is commonly overexpressed in neoplastic cells. GST P1-1 in the dog, *Canis lupus familiaris*, has merits as a marker for tumors and as a target for enzyme-activated prodrugs. We produced the canine enzyme CluGST P1-1 by heterologous bacterial expression and verified its cross-reactivity with antihuman-GST P1-1 antibodies. The catalytic activity with alternative substrates of biological significance was determined, and the most active substrate found was benzyl isothiocyanate. Among established GST inhibitors, Cibacron Blue showed positive cooperativity with an IC_50_ value of 43 nM. Dog GST P1-1 catalyzes activation of the prodrug Telcyta, but the activity is significantly lower than that of the human homolog.

## 1. Introduction

Glutathione transferases (GSTs) were discovered in mammalian liver tissues as cytosolic detoxication enzymes, conjugating electrophilic xenobiotics with glutathione and thereby facilitating their excretion [1]. Glutathione conjugates, of modest molecular size, can be converted to mercapturic acids and eliminated via the urine [2,3]. GSTs occur in multiple forms with distinct, but overlapping, substrate specificities, providing a molecular protective system against a wide variety of toxic agents. The substrates include epoxides, alkenals, quinones, and hydroperoxides, suggesting that GSTs have evolved as a guardrail against such toxic products that are formed ubiquitously and abundantly as byproducts of oxygen metabolism [4]. Both membrane-associated (MAPEG; [5]) and soluble [6] GSTs exist, and the main play-actors in mammalian detoxication appear to be members of the soluble GSTs of the Alpha, Mu, and Pi classes [7,8]. When tissues other than liver were investigated, the occurrence of the different GSTs was found to vary among organs and cell types, resulting in divergent capabilities for protection against toxic agents [9,10]. Particularly intriguing was the finding that the orthologs of the class Pi GST P1-1, abundantly expressed in placenta [11], were not found in human or rat liver—whereas, by contrast, two paralogs, GST P1-1 and GST P2-2, were found to occur in mouse liver [12]. The finding that the enzyme is overexpressed in certain neoplastic cells and tissues [13] was the basis for the proposal of using GST P1-1 as a tumor marker [14]. Furthermore, chemotherapeutic applications are based on targeting tumors overexpressing GST P1-1 by such prodrugs that are selectively activated by this enzyme [15].

Dogs are treasured domesticated animals as well as experimental models in pharmacological research. GSTs representing the classes responsible for detoxication of xenobiotics [7] were identified in both canine liver [16] and kidney [17]. In particular, the Pi class enzyme GST P1-1 was demonstrated in both of these organs, as well as in the lens [18]. Two GST P1-1 isoenzymes with identical N-terminal amino acid sequences and immunochemical reactivities were isolated from dog liver [16]. Differences in isoelectric points and chromatographic properties suggest that these two isoenzymes diverge by posttranslational processing. Canine GST P1-1, like the orthologs in other mammals, is overexpressed in certain cancers [17,19]. In view of its particular relationship to neoplasias and its potential to serve both as a tumor marker and a target for enzyme-activated chemotherapy, the current study provides further characterization of dog GST P1-1 with alternative substrates and inhibitors, as well as the ability to activate the anticancer prodrug Telcyta.

## 2. Results

### 2.1. Expression, Purification, and Characterization of Dog GST P1-1

**Figure 1:**
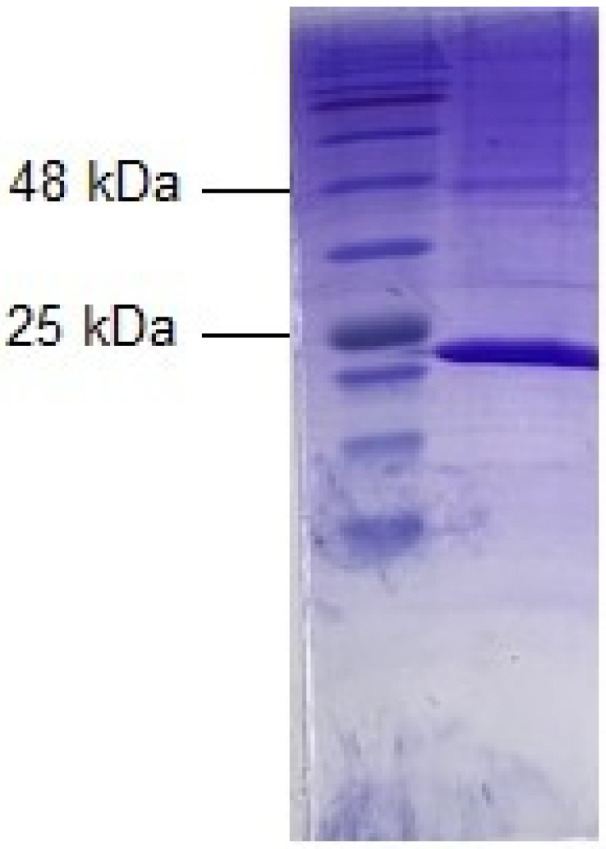
Analysis by SDS-PAGE of the purified dog GST P1-1 stained by Coomassie Brilliant Blue. The left lane shows molecular mass markers and the right lane the subunit of the purified enzyme.

For heterologous expression and facile isolation, DNA coding for CluGST P1-1 was synthesized with a codon usage optimized for high-level production in *Escherichia coli* and an N-terminal histidine tag for Ni-IMAC purification. A yield of 12 mg purified enzyme was obtained from 500 mL of bacterial culture medium and the protein was estimated to be >95% homogeneous, as judged by SDS-PAGE (Figure 1). The purified CluGST P1-1 was stored in aliquots at −20 °C.

A Western blot analysis was conducted to investigate cross-reactivity with antiserum raised in rabbits against human GST P1-1 [20]. The anti-HsaGST P1-1 antibodies gave a strong positive reaction with the dog GST P1-1.

### 2.2. Primary Structure

**Figure 2:**
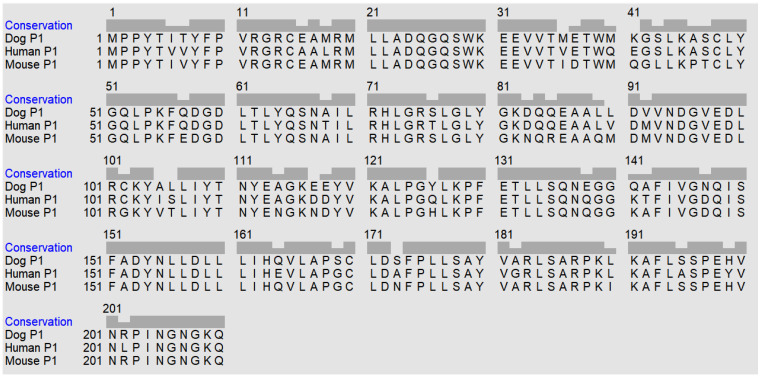
Alignment of dog, human, and mouse GST P1 sequences.

The *GSTP1* gene on chromosome 18 encoding the *Canis lupus familiaris* enzyme CluGST P1-1 (Gene ID: 476005) contains seven exons. The corresponding primary structure of the protein subunit GSTP1 is represented by 210 amino acid residues, which are 87.1% identical with the human and 87.6% identical with the mouse residues (Figure 2).

The calculated molecular mass of CluGSTP1 is 23,529 Da and the theoretical isoelectric point is 6.31. Assuming that the initiator Met is post-translationally eliminated in the mature protein, as found for other GST P1-1 proteins [21], the physiological values are 23,398 Da and 6.39, respectively.

Human GST P1-1 is a dimer and each of the identical subunits is composed of an N-terminal α/β domain and a C-terminal all-helical domain [22]. The N-capping box motif Ser150 -Xaa-Xaa-Asp153 [23], flanked by the (i – i + 5) hydrophobic staple motif Ile149 and Tyr154 [24], are conserved in the canine structure. These motifs occur at the N-terminus of the α6 helix in the protein core and were previously shown by mutagenesis to be important for folding and stability of human GST P1-1. Similarly, Tyr50, which operationally links a subunit to its neighbor in the catalytically active dimer [25], is a recurring feature in these related enzymes. Six residues—Tyr8, Arg14, Lys45, Gln52, Gln65, and Asp99—shown to functionally contribute to the G-site [26,27] are also conserved. Furthermore, Ser66, which forms a H-bond to the glutamyl moiety of glutathione, is also present in the canine enzyme.

### 2.3. Tertiary Structure

**Figure 3:**
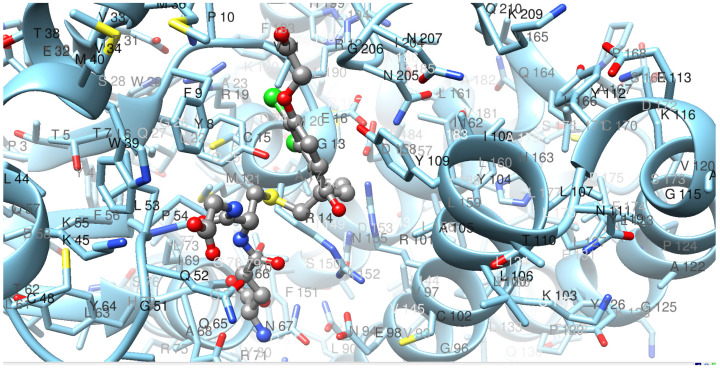
Structure of dog GST P1-1 with the conjugate of the substrate ethacrynic acid and glutathione (in color by element) bound to the active site. The model constructed in Modeller is based on the corresponding human crystal structure PDB ID: 11GS.

Considering the high sequence similarity among the Pi class GSTs, the tertiary structure of the dog GST P1-1 could be predicted by homology modeling. Figure 3 shows a predicted structure of one of the canine subunits based on human GST P1-1 in complex with the glutathione conjugate of the substrate ethacrynic acid. The ligand fits closely into the active-site cavity with the dichlorophenyl ring stacked between the aromatic residues Phe9 and Tyr109 in the H-site. This model is in accord with the similarity of catalytic activities for the human and dog enzymes found with ethacrynic acid as a substrate (see below).

### 2.4. Activities with Alternative Substrates

The purified dog GST P1-1 enzyme was assayed spectrophotometrically with various substrates representing different types of chemical reactions, including substitution, reduction, addition, and isomerization. The specific activities of the GST-class-distinguishing substrates ethacrynic acid, cumene hydroperoxide, and Δ^5^-androstene-3,17-dione were in the ranges originally used to distinguish members of mammalian classes [8]; in particular, a comparatively high value with ethacrynic acid and low values of the other two are considered a hallmark of the Pi class. The specific activities with most substrates were lower than the corresponding values previously determined for human GST P1-1 (Table 1). The highest activities of dog GST P1-1 were noted with 1-chloro-2,4-dinitrobenzene and benzyl isothiocyanate. Notably, although the specific activities of the dog and human enzymes were similar with the latter substrate, the dog GST P1-1 showed 10–20-fold lower values with the other four isothiocyanates (Table 1). The lowest specific activity of dog GST P1-1 was noted with Δ^5^-androstene-3,17-dione and low values were also obtained with cumene hydroperoxide and the trans-2-alkenals (Table 1).

### 2.5. Effects of Different GST Inhibitors

**Figure 4:**
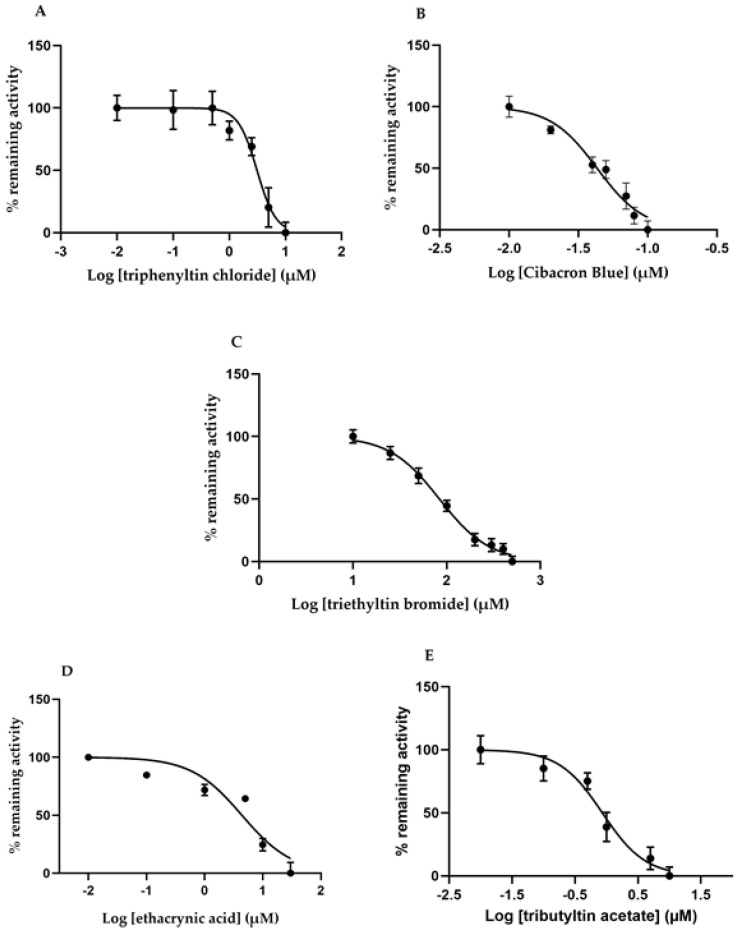
Inhibition of dog GST P1-1 with alternative compounds: (**A**) triphenyltin chloride, (**B**) Cibacron Blue, (**C**) triethyltin bromide, (**D**) Ethacrynic acid, and (**E**) tributyltin acetate. Remaining activity with the substrate benzyl isothiocyanate as compared with uninhibited enzyme plotted versus inhibitor concentration. Curves were fitted to the experimental data by nonlinear regression in GraphPad.

Compounds well-established as GST inhibitors [8] were tested with dog GST P1-1, using the most active substrate benzyl isothiocyanate in the catalyzed reactions. Enzyme activity was measured under standard conditions with 1 mM glutathione (GSH) and 0.4 mM benzyl isothiocyanate as substrates in the presence of different concentrations of the inhibitors (Figure 4). 

The shape of the curves indicated positive cooperativity with all inhibitors, except when ethacrynic acid (was tested with benzyl isothiocyanate as substrate). Similar deviations from simple hyperbolic inhibition curves have been found with other GSTs [29]. 

IC_50_ values determined under standard assay conditions spanned a range of three orders of magnitude (Table 2). Cibacron Blue was the most potent inhibitor (IC_50_ value of 43 nM), whereas the organotin compounds ranged between 0.85 and 83.6 µM, depending on the organic substituent. Ethacrynic acid—a diuretic drug which is also a substrate—showed an IC_50_ value of 4.38 µM. The latter compound was also tested with 1-chloro-2,4-dinitrobenzene as substrate to give an IC_50_ value of 17 µM.

### 2.6. Activation of the Prodrug Telcyta

**Figure 5:**
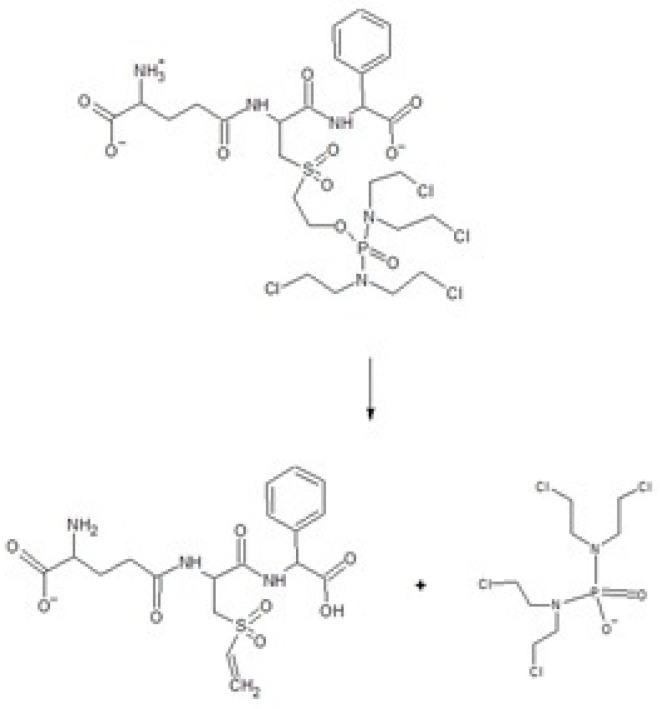
Activation of Telcyta catalyzed by GST P1-1. Both Telcyta (**top**) and the peptide product (**below**) are detectable by ninhydrin staining.

**Figure 6:**
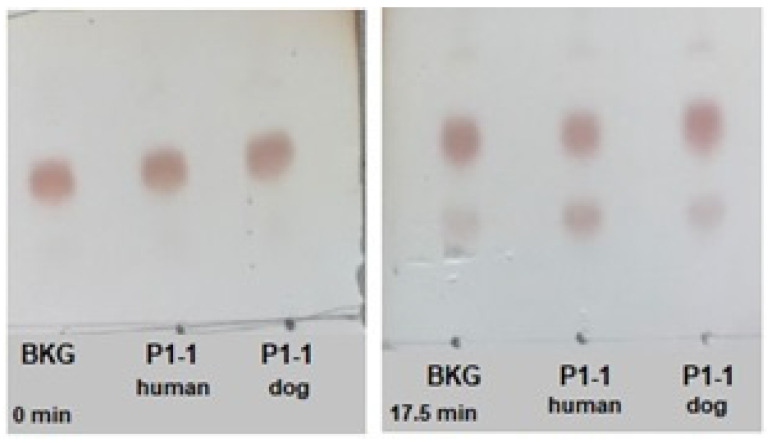
TLC analysis of the activation of Telcyta by human and dog GST P1-1. Aliquots of the reaction mixture were taken at a series of different time points. The left panel shows Telcyta at time zero and the right panel shows the emergence of the peptide product below after 17.5 min. Reactants were developed by ninhydrin staining and quantified; the nonenzymatic contribution (BKG) was subtracted from the reaction in the presence of 0.25 µg enzyme.

The prodrug Telcyta is a substrate for GSTs in a reaction releasing a multifunctional phosphorodiamidate mustard (Figure 5). The prodrug was most effectively activated by human GST P1-1 from the enzymes examined [30]. Telcyta was tested with the dog GST P1-1 for comparison. We developed a chromatographic method to assay the reaction. Aliquots of the reaction mixture were analyzed by thin-layer chromatography (TLC), and the ninhydrin-reactive product as well as the substrate were quantified at different time points (Figure 6). Conditions were chosen to ascertain a linear response of ninhydrin staining with increasing analyte concentration. The enzymatic activity was obtained by subtracting the nonenzymatic background reaction. Following this correction, the dog GST P1-1 was found to activate Telcyta, but only at 18-fold lower activity per mg of protein than the human enzyme did.

## 3. Discussion

The soluble mammalian GST proteins occur as dimers in their catalytically active form [31]. Some GST classes have similar members that can hybridize such that both homodimers and heterodimers occur [32]. It is therefore useful to designate the nature of the protein formed from the *GSTP1* gene as GST P1-1 to indicate its homodimeric structure composed of two identical P1 subunits [6]. The GST P1-1 sequences in mammalian tissues show a high degree of similarity. The primary structure of dog GST P1-1 contains four cysteine residues in positions 15, 48, 102, and 170, which are all conserved in the human enzyme (Figure 2). The mouse sequence has cysteines in the same positions except in 102, where a glycine is located. Examination of 97 GST P1-1 sequences in GenBank showed that cysteine residues are strictly conserved in positions 48 and 170. Furthermore, with the exception of the rodent *Fukomys damarensis* (Damaraland mole-rat), which contains glycine, all GST P1-1 sequences display Cys in position 15. In spite of the high degree of conservation, none of the cysteine residues appear essential for catalytic function, as judged by mutagenesis of the human GST P1-1 [33]. However, the sulfhydryl group of Cys48 shows an unusually low pKa value of 4.2, presumed to interact with Lys55 [34], which is also strictly conserved among the 97 GST P1-1 sequences. The dog GST P1-1 displays the Cys48–Lys55 pair, as well as the adjacent Tyr50, which is a conserved aromatic “key” residue (tyrosine or phenylalanine) fitting in a “lock” cavity in the neighboring subunit in the functional protein dimer [35]. Thus, the primary structure of CluGST P1-1 displays all the salient features of the members of the Pi class.

The structure of dog GST P1-1 obtained by homology modeling (Figure 3) shows the expected similarity with GST P1-1 structures from pig [36], human [22], and mouse [37]. Notwithstanding the resemblance, it is not clear why the human enzyme is approximately 10-fold more active than the dog enzyme with all isothiocyanates—except benzyl isothiocyanate—is, in spite of the similar specific activities with ethacrynic acid (Table 1). Mutagenesis of residue 105 in the human enzyme has demonstrated that the nature of the sidechain, which points toward the electrophilic substrate in the H-site, influences the catalytic activity [28]. Human GST P1-1/Ile105 has a bulkier residue than Ala105, which is found in dog GST P1-1.

A crystal structure of Cibacron Blue bound to human GST P1-1 has been published, but only the anthraquinone moiety of Cibacron Blue was visible and not the disulfophenyltriazine moiety [38]. Subsequent docking based on molecular force-field methods support the notion that the visible portion of Cibacron Blue accurately represents the interaction of this moiety of Cibacron Blue with the enzyme [39]. Our model of the dog GST P1-1 in complex with the inhibitor (not shown) indicates that the aromatic ring system of Cibacron Blue could make van der Waals contacts with Phe9, Val11, Ala105, Tyr109, and Gly206. The residues are the same as in the human enzyme with the exception of the presence of residue Ile105 in human GST P1-1. These interactions, in combination with a hydrogen bond between the sidechain of Tyr7 and a ring carbonyl group of Cibacron Blue, as well as an ionic interaction between the sulfonate group of Cibacron Blue and the guanidine group of Arg14, appear to contribute to the similarly tight binding of Cibacron Blue to dog GST P1-1.

Regarding the organotin compounds, a structure of triethyltin bromide as an inhibitor of the Alpha class GST A3-3 from horse was analyzed [40]. A corresponding homology model of the dog GST P1-1 in complex with triethyltin shows that the heavy atom can coordinate the oxygen of Tyr8, as well as the sulfur of glutathione, in a trigonal bipyramid structure similar to the one in the equine enzyme. Simultaneous blocking of the catalytically active Tyr8, as well as of the reactive atom of glutathione, appears instrumental in the inhibitory action of the organotin compounds.

Antibodies raised against human GST P1-1 were found to strongly cross-react with dog GST P1-1, attesting to their usefulness in immunohistochemistry applied to canine tissues and fluids. Originally proposed as a tumor marker, GST P1-1 has also been suggested as a biomarker of biological fluids in various clinical conditions [41].

**Figure 7:**
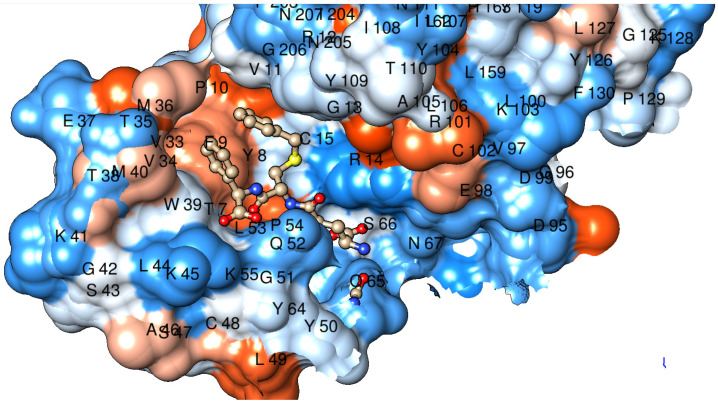
Structure of dog GST P1-1 in complex with TLK117, which features the same peptide backbone as Telcyta. The subunit of the enzyme is shown with atoms as spheres and the bound ligand in ball and stick representation. The benzyl group visible on the sulfur of TLK117 would be replaced by the bulky alkylating moiety in Telcyta. The model constructed in Modeller is based on the corresponding human crystal structure PDB ID: 10GS.

The notion that cancer cells often overexpress GST P1-1 was the basis for the design of prodrug inhibitors of the enzyme [30]. Telcyta (TLK286) was chosen as the main candidate for human clinical trials [42]. The molecule can be regarded as derived from the tripeptide glutathione with R(−)-phenylglycine replacing the C-terminal glycine residue and the sulfur oxidized to a sulfone in linkage with a tetravalent alkylating phosphorodiamidate mustard. A structure of a GST P1-1 in complex with Telcyta has not been solved, but a mechanism has been proposed which involves a central role of Tyr8 and a catalytic water molecule forming a network of interactions with the sulfone and a carboxyl group of TLK286 in the active site [43]. A structure of human GST P1-1 and γ-l-glutamyl-*S*-(benzyl)-l-cysteinyl-*R*(−)-phenylglycine (TLK117) has been determined by X-ray crystallography [44]. Figure 7 shows a model of dog GST P1-1 in complex with TLK117 based on homology with the human enzyme.

TLK117 features a R(−)-phenylglycine residue like Telcyta in the C-terminus of the peptide, and the model demonstrates that the residues Phe9, Met36, Trp39, and Met40 form a binding pocket accommodating the phenyl group of the unnatural amino acid. This phenyl group of the ligand is the characteristic feature that provides selectivity for GST P1-1 over other GSTs in which the substituent causes steric hindrance [30,44]. The peptide skeleton, including the R(−)-phenylglycine residue, is the same in Telcyta and TLK117, which can therefore be expected to bind in the same manner. In spite of this, and the conservation of the active-site Tyr8, the dog GST P1-1 was significantly less active than the human enzyme with Telcyta as a substrate. The bulky sidechain of Telcyta may not be positioned in a favorable pose for catalysis, which may be a reason for the lower activity of dog GST P1-1 as compared with human GST P1-1.

## 4. Materials and Methods

### 4.1. Heterologous Expression and Purification of Dog GST P1-1

DNA encoding the dog GST P1-1 enzyme (*Canis lupus familiaris*, NP_001239096.1) containing an N-terminal hexahistidine sequence was synthesized by ATUM (Newark, CA, USA). The DNA construct (pD444-NH expression vector, T5-His-ORF, ampicillin resistance) was extracted from the filter and used to transform *Escherichia coli* BL-21 (DE3) cells according to a standard heat shock protocol.

A starter culture of 5 mL lysogeny broth (LB) medium containing 2.5 mg ampicillin was inoculated with a single colony and grown overnight at 37 °C on a rotary shaker at 200 rpm. 5 mL culture was added to 500 mL of 2 TY expression medium together with 250 mg of ampicillin. The bacteria suspension was incubated at 200 rpm and 37 °C until an OD_600_ of 0.7 was reached. Isopropyl-β-d-1-thiogalactopyranoside (IPTG) was then added at 0.2 mM to induce dog GST P1-1 expression. The culture was allowed to grow for an additional three hours before cells were harvested by centrifugation at 7000× *g* and 4 °C for 5 min.

The bacterial pellet was resuspended in ice-cold Ni-IMAC binding buffer (20 mM sodium phosphate, 20 mM imidazole, 0.5 M NaCl, pH 7.4) with the addition of 0.2 mg of lysozyme (from chicken egg white) per mL, protease inhibitor cocktail, EDTA-free (1 tablet per 50 mL), and a final concentration of 0.2 mM tris(2-carboxyethyl)phosphine (TCEP). The bacterial suspension was sonicated and then centrifuged at 27,000× *g* and 4 °C for 30 min. The supernatant of the lysate was applied to a Ni-IMAC column (GE healthcare, His GraviTrap kit) and the affinity purification was carried out according to the manufacturer’s instructions.

The eluted GST P1-1 was dialyzed against 10 mM Tris-HCl, pH 7.8, 1 mM EDTA, and 0.2 mM TCEP. The protein concentration was determined by a NanoDrop spectrophotometer and the total amount of protein obtained from the purification was estimated to be 12.5 mg.

### 4.2. SDS-PAGE

The homogeneity of the purified dog GST P1-1 was evaluated by SDS-PAGE. Gels were prepared according to standard protocol (30% acrylamide, 0.8% bis-acrylamide) and molecular mass of the enzyme was estimated by a reference ladder (BLUeye prestained protein ladder) following Coomassie Brilliant Blue staining overnight.

### 4.3. Enzyme Activity Measurements

Activity measurements with alternative substrates were performed spectrophotometrically on a Shimadzu (UV-250 1 PC) instrument. All measurements were conducted at 30 °C and in a 1 mL quartz cuvette, except for the alkenals, which were measured in a 0.5 mL quartz cuvette with a 5 mm light path. The conditions used with the final concentrations in the cuvette were as follows. CDNB: (Δε_340 nm_ = 9600 cm^−1^ M^−1^), 1 mM GSH, 1 mM CDNB (dissolved in ethanol), measured at pH 6.5 in buffer A (0.1 M NaH_2_PO_4_/Na_2_HPO_4_ with 1 mM EDTA); CuOOH: (Δε_340 nm_ = −6200 cm^−1^ M^−1^), 1 mM GSH, 1.5 mM CuOOH (dissolved in acetonitrile), 0.1 mM NADPH, 0.3 units glutathione reductase, measured at pH 7.0 in buffer A; Alkenals: (Δε_225 nm_ = −19,200 cm^−1^ M^−1^), 0.5 mM GSH, 0.1 mM alkenals (dissolved in acetonitrile), measured at pH 6.5 in buffer A; EA: (Δε_270 nm_ = 5000 cm^−1^ M^−1^), 0.25 mM GSH, 0.2 mM EA (dissolved in acetonitrile), measured at pH 6.5 in buffer A; AD: (Δε_248 nm_ = 16,300 cm^−1^ M^−1^), 1 mM GSH, 0.1 mM AD (dissolved in methanol), measured at pH 8.0 in 0.025 M NaH_2_PO_4_/Na_2_HPO_4_ with 1 mM EDTA; PEITC: (Δε_274 nm_ = 8890 cm^−1^ M^−1^), BITC: (Δε_274 nm_ = 9250 cm^−1^ M^−1^), AITC: (Δε_274 nm_ = 7450 cm^−1^ M^−1^), PITC: (Δε_274 nm_ = 8350 cm^−1^ M^−1^), cHITC (Δε_274 nm_ = 8520 cm^−1^ M^−1^), 1 mM GSH, 0.4 mM ITC (dissolved in acetonitrile), measured at pH 6.5 in buffer A.

Inhibition studies were performed with 1 mM GSH and 0.4 mM benzyl isothiocyanate as substrates under standard assay conditions. Stock solutions of the inhibitors were made in ethanol, except for triphenyltin chloride, which was dissolved in acetone. Serial dilutions of all inhibitors were made in ethanol. The final ethanol concentration in the reaction system was 1% (*v*/*v*).

### 4.4. Thin-Layer Chromatography to Assay Activity with the Prodrug Telcyta

Telcyta is a tripeptide derivative, which releases an alkylating agent by the action of GSTs and is converted to another tripeptide. Both substrate and product peptides are reactive with ninhydrin and the reaction can be monitored by sampling of the reaction mixture at consecutive time points. The enzyme sample was prepared at a concentration of 0.5 mg/mL in 0.1 M NaH_2_PO_4_/Na_2_HPO_4_ buffer pH 7.5. Telcyta was dissolved in DMSO and diluted in the same buffer to a concentration of 6 mM. The reaction was started by mixing equal volumes of enzyme and Telcyta solutions to give final concentrations of 0.25 mg/mL and 3 mM, respectively, and allowed to proceed at 37 °C in a water bath. A background reaction was also measured in the absence of enzyme. Aliquots (1 µL) of the reaction mixture were taken at different time points and applied to TLC plates.

Six TLC plates (silica coated), one for each time point, were prepared and sampling occurred immediately after addition of Telcyta and every 3.5 min thereafter. The reaction was monitored over a total of 17.5 min. After reaction mixture was applied to each plate, all plates were dried at 37 °C for 10 min before being allowed to develop inside the chromatography jar. The mobile phase consisted of Milli-Q water, methanol, acetic acid, and ethyl acetate (ratio: 2:3:3:10). Once developed, plates were dried for another 10 min at 50 °C. For visualization, plates were dipped in ninhydrin solution (0.5% *w*/*v* in methanol) before incubation at 37 °C for 10 min.

Catalytic activity was estimated both by visual observation and by quantitative ImageJ analysis. Each TLC plate was photographed and the picture imported into ImageJ. The intensity and size of the stained areas, representing starting material and product, were quantified into peak areas. Time versus peak area was then plotted separately for starting material and product for each reaction mixture. The slope of the initial linear part of the progress curve was calculated as a measure of catalytic activity.

### 4.5. Modeling of Protein Structures

Molecular graphics and analyses were performed with UCSF Chimera, developed by the Resource for Biocomputing, Visualization, and Informatics at the University of California, San Francisco, with support from NIH P41-GM103311 [45]. Chimera interfaced to Modeller [46] was used for homology modeling of dog GST structures.

## 5. Conclusions

GST P1-1 has received special attention for various diagnostic purposes including immunohistochemistry and analysis of liquid specimens, as well as therapeutic applications in cancer. To date, research has focused on the human enzyme for possible medical purposes, but similar applications are relevant to veterinary medicine. The present investigation of the canine GST P1-1 demonstrates the extensive similarities with the human enzyme and lays a cornerstone for further biomedical research relevant to dogs.

## Figures and Tables

**Table 1:** Comparison of specific activities of dog GST P1-1 and human GST P1-1 with alternative substrates.

Specific Activity (µmol/min/mg)		
Substrate	Dog GST P1-1	Human GST P1-1/Ile105 ^1^
1-Chloro-2,4-dinitrobenzene (CDNB)	23.0 ± 1.3	106
Cumene hydroperoxide (CuOOH)	0.07 ± 0.01	0.03
Phenethyl isothiocyanate (PEITC)	6.37 ± 1.03	60
Benzyl isothiocyanate (BITC)	39.0 ± 5.2	63
Allyl isothiocyanate (AITC)	1.94 ± 0.60	38
Propyl isothiocyanate (PITC)	2.55 ± 0.20	49
Cyclohexyl isothiocyanate (cHITC)	1.27 ± 0.08	11
Ethacrynic acid (EA)	2.05 ± 0.13	2.0
trans-2-Pentenal	0.100 ± 0.002	N/A
trans-2-Nonenal	0.124 ± 0.005	N/A
trans-2-Decenal	0.111 ± 0.005	N/A
trans-2-Dodecenal	0.046 ± 0.006	N/A
Δ^5^-androstene-3,17-dione (AD)	0.014 ± 0.005	0.01

^1^ Data from [10,28]. N/A, not available.

**Table 2:** Effect of enzyme inhibitors on dog GST P1-1.

Inhibitor	IC_50_ (µM)
Ethacrynic acid	4.38 ± 1.96
Triethyltin bromide	83.6 ± 1.9
Triphenyltin chloride	3.11 ± 0.38
Tributyltin acetate	0.85 ± 0.13
Cibacron Blue	0.043 ± 0.007

## Data Availability

Original data available from the authors.

## Abbreviations

GSH	Glutathione
GST	Glutathione transferases
Clu	Canis lupus familiaris
CDNB	1-Chloro-2,4-dinitrobenzene
PEITC	Phenethyl Isothiocyanate
BITC	Benzyl isothiocyanate
AITC	Allyl isothiocyanate
PITC	Propyl isothiocyanate
cHITC	Cyclohexyl isothiocyanate
CuOOH	Cumene hydroperoxide
EA	Ethacrynic acid
AD	Δ^5^-Androstene-3,17-dione
Telcyta (TLK286)	γ-l-Glutamyl-α-amino-β-(2-ethyl-*N*,*N*,*N*′,*N*′-tetrakis(2-chloroethyl)phosphorodiamidate)sulfonylpropionyl-*R*(−)phenylglycine
TLK117	γ-l-Glutamyl-*S*-(benzyl)-l-cysteinyl-*R*(−)-phenylglycine

## References

[1] Josephy P.D., Mannervik B. (2006). Molecular Toxicology.

[2] Boyland E., Chasseaud L.F. (1969). The role of glutathione and glutathione S-transferases in mercapturic acid biosynthesis. Adv. Enzymol. Relat. Areas Mol. Biol..

[3] Hanna P.E., Anders M.W. (2019). The mercapturic acid pathway. Crit. Rev. Toxicol..

[4] Mannervik B. (1986). Glutathione and the Evolution of Enzymes for Detoxication of Products of Oxygen-Metabolism. Chem. Scr..

[5] Jakobsson P.J., Morgenstern R., Mancini J., Ford-Hutchinson A., Persson B. (1999). Common structural features of MAPEG—A widespread superfamily of membrane associated proteins with highly divergent functions in eicosanoid and glutathione metabolism. Protein Sci..

[6] Mannervik B., Board P.G., Hayes J.D., Listowsky I., Pearson W.R. (2005). Nomenclature for mammalian soluble glutathione transferases. Methods Enzymol..

[7] Tan H.M., Low W.Y. (2018). Rapid birth-death evolution and positive selection in detoxification-type glutathione S-transferases in mammals. PLoS ONE.

[8] Mannervik B., Ålin P., Guthenberg C., Jensson H., Tahir M.K., Warholm M., Jörnvall H. (1985). Identification of three classes of cytosolic glutathione transferase common to several mammalian species: Correlation between structural data and enzymatic properties. Proc. Natl. Acad. Sci. USA.

[9] Mannervik B., Guthenberg C., Jensson H., Warholm M., Ålin P., Larsson A., Orrenius S., Holmgren A., Mannervik B. (1983). Isozymes of Glutathione S-Transferases in Rat and Human Tissues. Functions of Glutathione: Biochemical, Physiological, Toxicological, and Clinical Aspects.

[10] Mannervik B., Guthenberg C., Jensson H., Tahir M.K., Warholm M., Ålin P., Paton W., Mitchell J.F., Turner P. (1984). Species and Tissue Differences in the Occurrence of Multiple Forms of Rat and Human Glutathione Transferases. Proceedings of the IUPHAR 9th International Congress of Pharmacology.

[11] Guthenberg C., Åkerfeldt K., Mannervik B. (1979). Purification of glutathione-S-transferase from human placenta. Acta Chem. Scand. B.

[12] Bammler T.K., Smith C.A., Wolf C.R. (1994). Isolation and characterization of two mouse Pi-class glutathione S-transferase genes. Biochem. J..

[13] Mannervik B., Castro V.M., Danielson U.H., Tahir M.K., Hansson J., Ringborg U. (1987). Expression of class Pi glutathione transferase in human malignant melanoma cells. Carcinogenesis.

[14] Sato K. (1989). Glutathione transferases as markers of preneoplasia and neoplasia. Adv. Cancer Res..

[15] Lyttle M.H., Hocker M.D., Hui H.C., Caldwell C.G., Aaron D.T., Engqvist-Goldstein A., Flatgaard J.E., Bauer K.E. (1994). Isozyme-specific glutathione-S-transferase inhibitors: Design and synthesis. J. Med. Chem..

[16] Igarashi T., Kohara A., Shikata Y., Sagami F., Sonoda J., Horie T., Satoh T. (1991). The unique feature of dog liver cytosolic glutathione S-transferases. An isozyme not retained on the affinity column has the highest activity toward 1,2-dichloro-4-nitrobenzene. J. Biol. Chem..

[17] Bohets H.H., Nouwen E.J., De Broe M.E., Dierickx P.J. (1996). The cytosolic glutathione S-transferase isoenzymes in the dog kidney cortex as compared with the corresponding MDCK renal cell line. Biochim. Biophys. Acta.

[18] Nishinaka T., Kodaka R., Nanjo H., Terada T., Mizoguchi T., Nishihara T. (1992). Purification and characterization of glutathione S-transferase isozymes in dog lens. Int. J. Biochem..

[19] Gerardi D.G., Tinucci-Costa M., Silveira A.C.T., Moro J.V. (2014). Expression of P-glycoprotein, multidrug resistance-associated protein, glutathione-S-transferase pi and p53 in canine transmissible venereal tumor. Pesqui. Vet. Bras..

[20] Hao X.Y., Castro V.M., Bergh J., Sundström B., Mannervik B. (1994). Isoenzyme-specific quantitative immunoassays for cytosolic glutathione transferases and measurement of the enzymes in blood plasma from cancer patients and in tumor cell lines. Biochim. Biophys. Acta.

[21] Mannervik B., Danielson U.H. (1988). Glutathione transferases--structure and catalytic activity. CRC Crit. Rev. Biochem..

[22] Reinemer P., Dirr H.W., Ladenstein R., Huber R., Lo Bello M., Federici G., Parker M.W. (1992). Three-dimensional structure of class pi glutathione S-transferase from human placenta in complex with S-hexylglutathione at 2.8 A resolution. J. Mol. Biol..

[23] Dragani B., Stenberg G., Melino S., Petruzzelli R., Mannervik B., Aceto A. (1997). The conserved N-capping box in the hydrophobic core of glutathione S-transferase P1-1 is essential for refolding. Identification of a buried and conserved hydrogen bond important for protein stability. J. Biol. Chem..

[24] Stenberg G., Dragani B., Cocco R., Mannervik B., Aceto A. (2000). A conserved “hydrophobic staple motif” plays a crucial role in the refolding of human glutathione transferase P1-1. J. Biol. Chem..

[25] Hegazy U.M., Mannervik B., Stenberg G. (2004). Functional role of the lock and key motif at the subunit interface of glutathione transferase p1-1. J. Biol. Chem..

[26] Kolm R.H., Sroga G.E., Mannervik B. (1992). Participation of the phenolic hydroxyl group of Tyr-8 in the catalytic mechanism of human glutathione transferase P1-1. Biochem. J..

[27] Widersten M., Kolm R.H., Björnestedt R., Mannervik B. (1992). Contribution of five amino acid residues in the glutathione-binding site to the function of human glutathione transferase P1-1. Biochem. J..

[28] Johansson A.S., Stenberg G., Widersten M., Mannervik B. (1998). Structure-activity relationships and thermal stability of human glutathione transferase P1-1 governed by the H-site residue 105. J. Mol. Biol..

[29] Lindström H., Mazari A.M.A., Musdal Y., Mannervik B. (2019). Potent inhibitors of equine steroid isomerase EcaGST A3-3. PLoS ONE.

[30] Lyttle M.H., Satyam A., Hocker M.D., Bauer K.E., Caldwell C.G., Hui H.C., Morgan A.S., Mergia A., Kauvar L.M. (1994). Glutathione-S-transferase activates novel alkylating agents. J. Med. Chem..

[31] Fabrini R., De Luca A., Stella L., Mei G., Orioni B., Ciccone S., Federici G., Lo Bello M., Ricci G. (2009). Monomer-dimer equilibrium in glutathione transferases: A critical re-examination. Biochemistry.

[32] Mannervik B., Jensson H. (1982). Binary combinations of four protein subunits with different catalytic specificities explain the relationship between six basic glutathione S-transferases in rat liver cytosol. J. Biol. Chem..

[33] Hegazy U.M., Tars K., Hellman U., Mannervik B. (2008). Modulating catalytic activity by unnatural amino acid residues in a GSH-binding loop of GST P1-1. J. Mol. Biol..

[34] Ricci G., Lo Bello M., Caccurri A.M., Pastore A., Nuccetelli M., Parker M.W., Federici G. (1995). Site-directed mutagenesis of human glutathione transferase P1-1. Mutation of Cys-47 induces a positive cooperativity in glutathione transferase P1-1. J. Biol. Chem..

[35] Stenberg G., Abdalla A.M., Mannervik B. (2000). Tyrosine 50 at the subunit interface of dimeric human glutathione transferase P1-1 is a structural key residue for modulating protein stability and catalytic function. Biochem. Biophys. Res. Commun..

[36] Reinemer P., Dirr H.W., Ladenstein R., Schäffer J., Gallay O., Huber R. (1991). The three-dimensional structure of class pi glutathione S-transferase in complex with glutathione sulfonate at 2.3 A resolution. EMBO J..

[37] García-Sáez I., Párraga A., Phillips M.F., Mantle T.J., Coll M. (1994). Molecular structure at 1.8 A of mouse liver class pi glutathione S-transferase complexed with S-(p-nitrobenzyl)glutathione and other inhibitors. J. Mol. Biol..

[38] Oakley A.J., Lo Bello M., Nuccetelli M., Mazzetti A.P., Parker M.W. (1999). The ligandin (non-substrate) binding site of human Pi class glutathione transferase is located in the electrophile binding site (H-site). J. Mol. Biol..

[39] Al-Qattan M.N., Mordi M.N., Mansor S.M. (2016). Assembly of ligands interaction models for glutathione-S-transferases from Plasmodium falciparum, human and mouse using enzyme kinetics and molecular docking. Comput. Biol. Chem..

[40] Škerlová J., Ismail A., Lindström H., Sjödin B., Mannervik B., Stenmark P. (2021). Structural and functional analysis of the inhibition of equine glutathione transferase A3-3 by organotin endocrine disrupting pollutants. Environ. Pollut..

[41] Bocedi A., Noce A., Marrone G., Noce G., Cattani G., Gambardella G., Di Lauro M., Di Daniele N., Ricci G. (2019). Glutathione Transferase P1-1 an Enzyme Useful in Biomedicine and as Biomarker in Clinical Practice and in Environmental Pollution. Nutrients.

[42] Kavanagh J.J., Gershenson D.M., Choi H., Lewis L., Patel K., Brown G.L., Garcia A., Spriggs D.R. (2005). Multi-institutional phase 2 study of TLK286 (TELCYTA, a glutathione S-transferase P1-1 activated glutathione analog prodrug) in patients with platinum and paclitaxel refractory or resistant ovarian cancer. Int. J. Gynecol. Cancer.

[43] Dourado D.F., Fernandes P.A., Ramos M.J., Mannervik B. (2013). Mechanism of glutathione transferase P1-1-catalyzed activation of the prodrug canfosfamide (TLK286, TELCYTA). Biochemistry.

[44] Oakley A.J., Lo Bello M., Battistoni A., Ricci G., Rossjohn J., Villar H.O., Parker M.W. (1997). The structures of human glutathione transferase P1-1 in complex with glutathione and various inhibitors at high resolution. J. Mol. Biol..

[45] Pettersen E.F., Goddard T.D., Huang C.C., Couch G.S., Greenblatt D.M., Meng E.C., Ferrin T.E. (2004). UCSF Chimera—A visualization system for exploratory research and analysis. J. Comput. Chem..

[46] Sali A., Blundell T.L. (1993). Comparative protein modelling by satisfaction of spatial restraints. J. Mol. Biol..

